# Multi-Patterned Dynamics of Mitochondrial Fission and Fusion in a Living Cell

**DOI:** 10.1371/journal.pone.0019879

**Published:** 2012-05-23

**Authors:** Shiqi Wang, Weiming Xiao, Sicong Shan, Chunsun Jiang, Ming Chen, Yan Zhang, Shouqin Lü, Juan Chen, Chuanmao Zhang, Quan Chen, Mian Long

**Affiliations:** 1 Key Laboratory of Microgravity (National Microgravity Laboratory), Chinese Academy of Sciences, Beijing, China; 2 Center for Biomechanics and Bioengineering, Institute of Mechanics, Chinese Academy of Sciences, Beijing, China; 3 State Key Laboratory of Biomembrane and Membrane Biotechnology, Institute of Zoology, Chinese Academy of Sciences, Beijing, China; 4 National Key Laboratory of Biomembrane and Membrane Biotechnology and Department of Cell Biology and Genetics, College of Life Sciences, Peking University, Beijing, China; 5 Institute of Vertebrate Paleontology and Paleoanthropology, Chinese Academy of Sciences, Beijing, China; University of Colorado, Boulder, United States of America

## Abstract

Mitochondria are highly-dynamic organelles, but it is challenging to monitor quantitatively their dynamics in a living cell. Here we developed a novel approach to determine the global occurrence of mitochondrial fission and fusion events in living human epithelial cells (Hela) and mouse embryonic fibroblast cells (MEF). Distinct patterns of sequential events including fusion followed by fission (*Fu-Fi*), the so-called “kiss and run” model previously described, fission followed by fusion (*Fi-Fu*), fusion followed by fusion (*Fu-Fu*), and fission followed by fission (*Fi-Fi*) were observed concurrently. The paired events appeared in high frequencies with short lifetimes and large sizes of individual mitochondria, as compared to those for unpaired events. The high frequencies of paired events were found to be biologically significant. The presence of membrane uncoupler CCCP enhanced the frequency of paired events (from both *Fu-Fi* and *Fi-Fu* patterns) with a reduced mitochondrial size. Knock-out of mitofusin protein Mfn1 increased the frequency of fission with increased lifetime of unpaired events whereas deletion of both Mfn1 and Mfn2 resulted in an instable dynamics. These results indicated that the paired events were dominant but unpaired events were not negligible, which provided a new insight into mitochondrial dynamics. In addition to kiss and run model of action, our data suggest that, from a global visualization over an entire cell, multiple patterns of action appeared in mitochondrial fusion and fission.

## Introduction

In a living cell, mitochondria tend to form a highly-interconnected network and undergo continuous movements, fission, and fusion [1]–[2]. Such a complex and dynamic network is crucial to regulate cellular energy metabolism, organelle distribution and biogenesis, and cell apoptosis. For example, mitochondrial fission is important to distribute the organelles inside a cell and manipulates their biological activities at local sites [3]. By contrast, mitochondrial fusion forms continuous membranes and matrix lumen to implement the transportation of solutes, metabolites, and proteins and the regulation of membrane potential [4]. Several GTPases in dynamin family are known to regulate mitochondrial fission and fusion [1], [5]–[7]. Meanwhile, mitochondrial movement appears to be a directed motion along the cytoskeleton [8]–[11] since the mutation and inhibition of cytoskeletal proteins affect the movement. Evidently, both the regulating mechanisms at subcellular or molecular level and the dynamics of mitochondrial movements are important to cellular biological functions.

While the regulating mechanisms of mitochondrial fission and fusion have been extensively studied at a molecular level, only a few works are focused on understanding mitochondrial dynamics at a subcellular level. Several models were proposed to describe the dynamical and mechanical features of mitochondrial movement, fission, and fusion in yeast or in mammalian systems [11]–[20]. For example, a stochastic diffusion model was developed to describe the movement of a single mitochondrion by superposing the stochastic fluctuation onto the directed motion [9]. Fractal property of moving trajectory of subcellular vesicles [11] and three-dimensional (3D) movement of cytoplasmic membrane vesicles [12] were visualized experimentally. Moreover, one-dimensional (1D) movement of mitochondria in axons was quantified and the spatial distribution of mitochondria was found to follow a Poisson distribution [13], [14]. While most of those analyses for mitochondrial dynamics were performed in fission or budding yeast [15], [16] or in yeast during meiosis [17], it is difficult to quantitatively monitor mitochondrial fission and fusion in a living eukaryotic cell [18]. For example, an important issue is how to define the balance between fission and fusion events since the enhancement or reduction in the quantity of individual mitochondria may bias the estimation of fission or fusion rate. Thus, new approaches are required to monitor the occurrence of fission and fusion events and then to define the mitochondrial geometry and dynamics in a living cell.

Recently, a dynamic model of paired consecutive events was proposed where a fusion event was assumed to trigger a sequential fission event in a living cell [4]. Such the dynamics was biologically significant since the membrane potential of two daughter mitochondria was altered and the depolarized mitochondria enhanced their autophagy when triggered sequentially. A “kiss and run” pattern was characterized in the life cycle of mitochondria where fusion triggers fission but fission has no effect on the timing of following fusion. In a parallel work, this model was further developed by visualizing a transient fusion event that exhibited rapid kinetics of the sequential and separable mergers of the outer and inner membranes but allowed only partial exchange of integral membrane proteins [21]. It still remains unknown, however, whether there exist the distinct patterns of consecutive fusion and fission events and what are the underlying morphological and dynamic changes of those sequential actions. Here we developed a novel approach to visualize the time course of consecutive fusion and fission and to measure quantitatively the mitochondrial dynamics in living Hela and MEF cells. An evolution roadmap of mitochondrial geometry was presented concurrently in a living cell. We found that the mitochondrial dynamics was highly correlated with their functions in respiratory uncoupling and microtubule depolymerization, and with the expression of mitofusin proteins.

## Results

### Mitochondria evolve dynamically in a living cell

**Figure 1:**
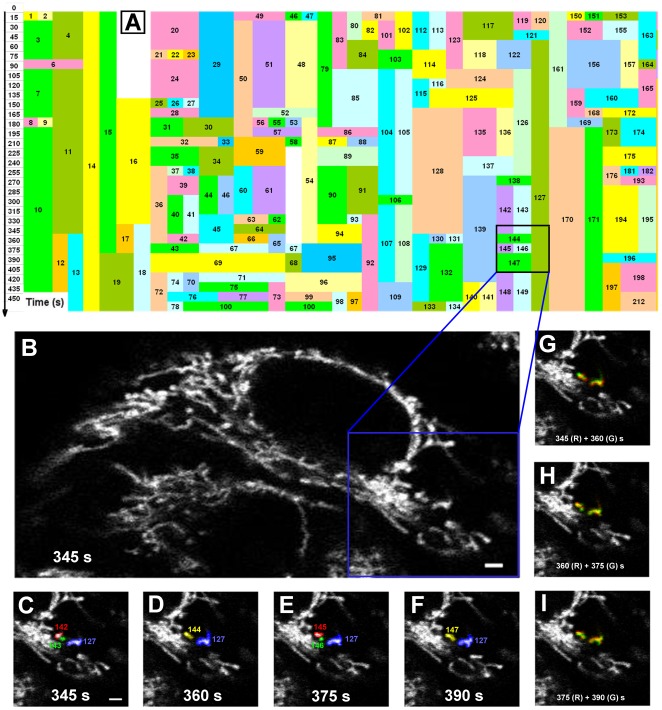
Mitochondrial evolution in a living Hela cell. A global roadmap of mitochondrial evolution was presented up to 450 s (*A*) for an entire Hela cell (*B*) where distinct mitochondria were denoted as *numbers* in different *colored* boxes. Time-lapsed images obtained from a dual-photon laser scanning microscopy were illustrated at *t* = 345 (*C*), 360 (*D*), 375 (*E*), and 390 (*F*) s for those typical mitochondria located at right lower region of the cell (*black box* in *A* and *blue box* in *B*) and colored with *numbers*. Also plotted were merged images at two sequential time points where all the mitochondria were colored as *red* (*R*) at a given point and as *green* (*G*) at the following point (*G*–*I*). *Bar* = 2 µm.

Mitochondria are highly-dynamic and undergo continuous movement, fission, and fusion. In the current work, we employed a time-lapsed confocal microscopy to monitor individual mitochondria in a human epithelial carcinoma (Hela) cell (Movie S1) or a wild-type mouse embryonic fibroblast (MEF-WT) cell (Movie S2). Over 90% of mitochondria were globally counted for an entire cell spanning over observation duration and all the fusion and fission events for those identified mitochondria were determined one-by-one using digitalized images. To assure that the fusion or fission events so observed are convincing, 3D reconstruction of the images was done for each mitochondrion (Fig. S1) and time-lapsed dynamics was monitored for further quantitative analysis (Movie S3). Here a fusion event was defined when at least two individual mitochondria reserved physical contact to form a single mitochondrion while a fission event was determined when a single mitochondrion was physically separated into at least two mitochondria. Applying this new approach resulted in a roadmap of mitochondrial evolution (Fig. 1*A*). In a typical measurement for a Hela cell (Fig. 1*B*), #127 mitochondrion identified at the beginning of observation (*t* = 345 s) survived as an isolated organelle over entire time span, whereas #142 and #143 mitochondria, also identified at *t* = 345 s, fused as a single #144 mitochondrion at *t* = 360 s. The fused organelle further fragmented into #145 and #146 mitochondria at *t* = 375 s, which reunited together as #147 mitochondrion at *t* = 390 s (*colored* with *numbers* in Figs. 1*C*–*F*). Merged images at two sequential time points also demonstrated the dynamic evolution (Figs. 1*G*–*I*). Such the roadmap not only identified each fission or fusion event of an isolated mitochondrion, but it also supported that mitochondrial fusion and fission were highly dynamic along different pathways.

Our approach was further confirmed by the conventionally biological measurements in mitochondrial analyses. We first compared the images between the original segmentation from confocal microscopy (*left column*) and the binarized segmentation (*right column*) (Fig. S2). It was found that both types of images are consistent with each other. We also compared the identification of individual mitochondria and true fusion or fission events between photoactivated GFP measurements and 3D reconstruction protocol reported here (Figs. S3 and S4). Here *green color* clearly indicates single mitochondria, which are also identified by the binarized images. A fusion event is also visualized by *yellow color* (mixed with *green* and *red colors*) shown on the merged image, which is also observed by the binarized images. Similar results were obtained from >10 time independent measurements, implying that our approach worked well with the identical recognition of individual objects and dynamic events. Noting that photoactivated GFP measurements are mainly focusing on those regional events inside a living cell, our approach is assured to visualize the true mitochondrial fusion or fission events and to estimate the global dynamics cross entire cell.

### Distinct patterns exist in mitochondria dynamics

**Figure 2:**
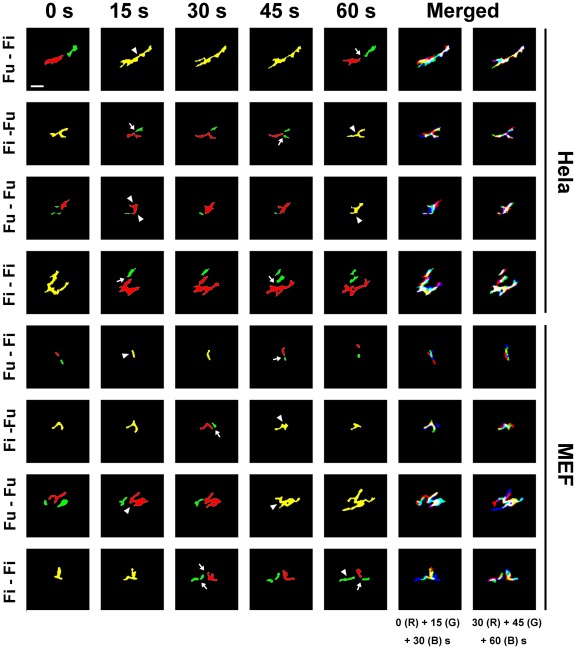
Sequential events of mitochondrial fission and fusion in a Hela or MEF cell. Time-lapsed images were employed to illustrate four patterns of sequential events of mitochondrial fission and fusion in a Hela (*1^st^*–*4^th^ rows*) or a MEF (*5^th^*–*8^th^ rows*) cell, that is, fusion followed by fission (*Fu-Fi*, *1^st^* and *5^th^ rows*), fission followed by fusion (*Fi-Fu*, *2^nd^* and *6^th^ rows*), fusion followed by fusion (*Fu-Fu*, *3^rd^* and *7^th^ rows*), and fission followed by fission (*4^th^* and *8^th^ rows*), at *t* = 0 (*1^st^ column*), 15 (*2^nd^ column*), 30 (*3^rd^ column*), 45 (*4^th^ column*), and 60 (*5^th^ column*) s. In the panels, an isolated mitochondrial was colored as *red*, *green*, or *blue*, and *arrows* denoted as a fission event and *arrow heads* indicated a fusion event. Also plotted were merged images at three sequential time points where all the mitochondria were colored as *red* (*R*) at a given point, as *green* (*G*) at the following point, and as *blue* (*B*) at the last point (6^th^ and 7^th^ columns). *Bar* = 1 µm.

We further analyzed the consecutive fusion or fission event upon the resulted evolution roadmap of individual mitochondria. Interestingly, distinct patterns for sequential events were found to be present concurrently in a Hela cell, that is, fusion followed by fission (*Fu-Fi*), fission followed by fusion (*Fi-Fu*), fusion followed by fusion (*Fu-Fu*), and fission followed by fission (*Fi-Fi*) (Fig. 2 and Movie S1). This first pattern is similar to the one that is previously defined as a “kiss and run” model [21] and accordingly, the other three refer to as, namely, “run and kiss”, “kiss and kiss”, and “run and run” patterns. In a typical *Fu-Fi* pattern, for example, two isolated mitochondria fused into a single mitochondrion at *t* = 15 s, which subsequently followed by fragmentation into two new individual mitochondria at *t* = 60 s (1^st^ *row*). A branched mitochondrion segregated into two small-sized mitochondria at *t* = 45 s and re-fused, together with another mitochondria, into a single vesicle at *t* = 60 s (2^nd^ *row*), indicating a *Fi-Fu* pattern. A *Fu-Fu* or *Fi-Fi* pattern was visualized as two sequential fusion events at *t* = 15 and 60 s (3^rd^ *row*) or as two sequential fission events at *t* = 15 and 45 s (4^th^ *row*). Similar types of distinct patterns were also observed at different time points in a MEF-WT cell (Fig. 2 and Movie S2), demonstrating that all of four patterns are present in distinct cell lineages. It was also noted that the occurrence of sequential fission and fusion events took place only for those adjacent mitochondria geometrically possible to have physical contact that is confirmed in both two-dimensional (2D) plane and 3D reconstruction.

**Figure 3:**
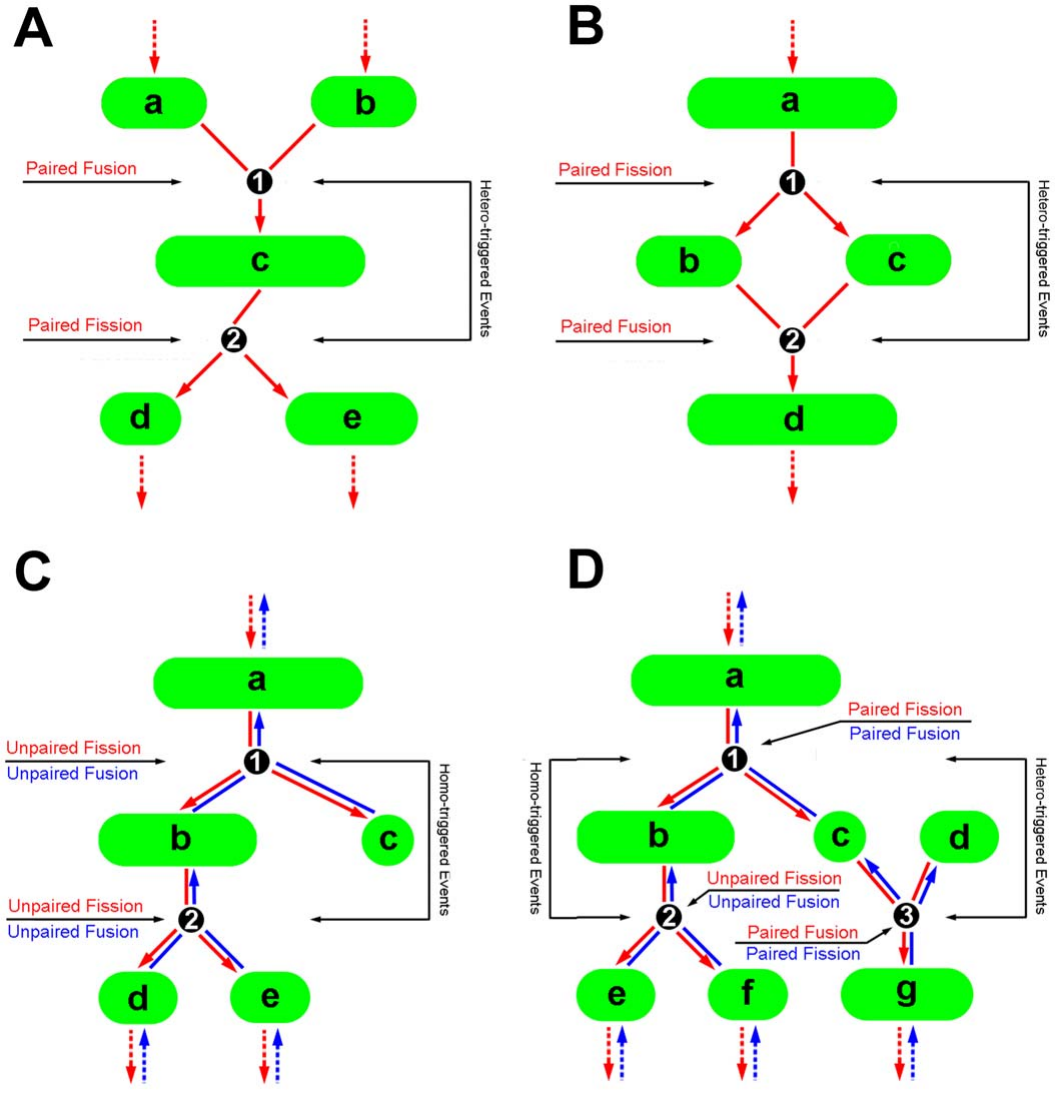
Schematic of different dynamic patterns of mitochondrial fission and fusion. Definitions of hetero- (*A*, *B*), homo- (*C*, *D*) triggered patterns and of paired or unpaired events (*A–D*). Here a *letter* was denoted as a mitochondrion and a *number* was referred to an event.

**Figure 4:**
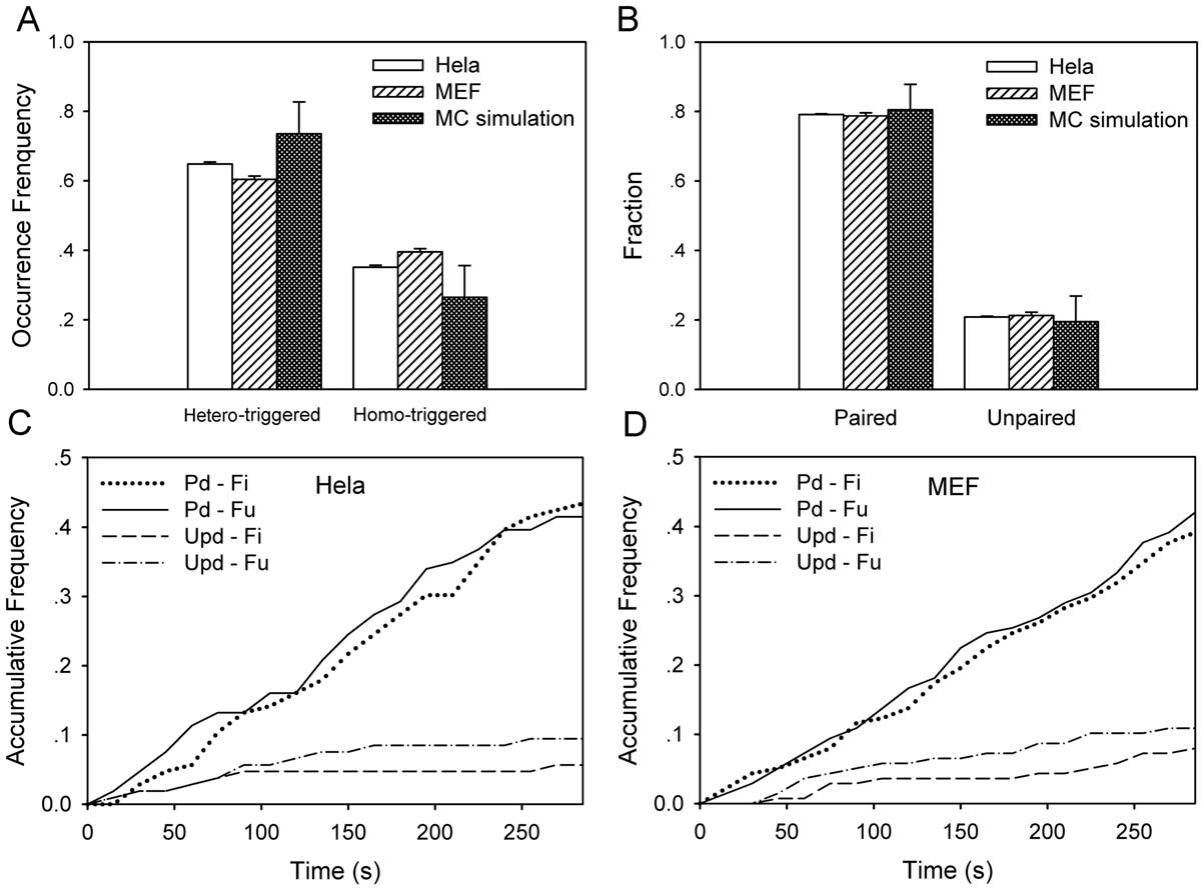
Multi-dynamic patterns in a Hela or MEF cell or from simulations. Comparisons of occurrence frequency of distinct patterns in a Hela (*A*) and fraction of paired and unpaired events (*B*) in a Hela (*open bars*) or MEF (*hatched bars*) cell as well as from Monte-Carlo simulations (*solid bars*). Also plotted were the time courses of accumulative frequency of paired (*Pd*) or unpaired (*UPd*) fission (*Fi*) and fusion (*Fu*) events in a Hela (*C*) or MEF (*D*) cell up to *t* = 285 s. Data were presented as mean ± standard error (SE) measured from total 21–43 mitochondria and 121–231 events per cell.

The evolution roadmap obtained (Fig. 1) also enables one to estimate the occurrence or accumulative frequency of a fusion or fission event and the lifetime and size of individual mitochondria (seen in the Materials and Methods). Here we further quantified the presence of distinct patterns using an occurrence frequency, defined as the occurrence fraction of sequential events for that pattern over entire duration (Fig. 3). Here a “hetero-triggered” event was determined when two mitochondria fused into a mitochondrion followed by its fission into two new mitochondria (Fig. 3*A*) or when a single mitochondrion fragmented into two mitochondria followed by its fusion into a new mitochondrion (Fig. 3*B*). A “homo-triggered” event was denoted as a mitochondrion fragmented into two mitochondria followed by a sequential fission into new mitochondria or *vise versa* (*red* and *blue lines* in Figs. 3*C*–3*D*). Note that the reason we define the homo- or hetero-triggered event, in addition to the above four patterns, is attempting to further look at the coupling of two consecutive events. It was found that the average frequency yielded a definite value and was not negligible in all four patterns either for a Hela (0.17–0.41) or MEF (0.18–0.42) cell. It was also indicated that the occurrence was not equivalent among distinct patterns where the frequency for *Fi-Fu* pattern was the highest in both cell lines (Table 1). Moreover, the occurrence frequency of hetero-triggered pattern (sum of frequencies for *Fu-Fi* and *Fi-Fu* patterns) was significantly higher than that of homo-triggered events (sum of frequencies for *Fu-Fu* and *Fi-Fi*), that is, 0.65 and 0.35 for a Hela cell (*open bars*, *P* = 0.001) and 0.60 and 0.40 for a MEF cell (*hatched bars*, *P* = 0.004) (Fig. 4*A*), suggesting that the occurrence of hetero-triggered pattern was dominant in mitochondrial dynamics.

**Table 1:** Summaries of mitochondrial dynamics in intact or treated Hela and MEF cells.

	Occurrence frequency of sequential events				Fraction of paired and unpaired events				Normalized lifetime		Normalized size	
	*Fu-Fi*	*Fi-Fu*	*Fu-Fu*	*Fi-Fi*	*Pd-Fi*	*Pd-Fu*	*UPd-Fi*	*UPd-Fu*	*Pd*	*UPd*	*Pd*	*UPd*
^ζ^Hela cell												
Intact	0.24±0.02	0.41±0.01	0.17±0.01	0.18±0.02	0.39±0.00	0.40±0.00	0.11±0.01	0.10±0.01	0.04±0.01	0.12±0.00	0.28±0.09	0.10±0.03
	(94)	(158)	(67)	(69)	(113)	(114)	(32)	(28)	{223}	{291}	{223}	{291}
+CCCP	0.30±0.03	0.42±0.00	0.12±0.01	0.16±0.0	0.45±0.01	0.42±0.00	0.08±0.01	0.05±0.00	0.05±0.00	0.11±0.01	0.19±0.02	0.07±0.00
	(122)	(185)	(60)	(82)	(158)	(148)	(26)	(19)	{299}	{286}	{299}	{286}
+Noco.	0.25±0.01	0.38±0.01	0.19±0.01	0.18±0.01	0.42±0.00	0.43±0.00	0.07±0.00	0.08±0.01	0.04±0.01	0.10±0.04	0.32±0.09	0.12±0.05
	(115)	(180)	(88)	(84)	(141)	(144)	(24)	(25)	{263}	{370}	{263}	{370}
^ζ^MEF cell												
WT	0.18±0.00	0.42±0.01	0.22±0.01	0.18±0.00	0.40±0.01	0.39±0.02	0.07±0.01	0.14±0.02	0.07±0.00	0.13±0.00	0.35±0.05	0.14±0.05
	(109)	(259)	(135)	(109)	(141)	(141)	(27)	(48)	{255}	{305}	{255}	{305}
Mfn1 −/−	0.24±0.01	0.43±0.01	0.19±0.01	0.14±0.00	0.37±0.01	0.36±0.01	0.12±0.00	0.15±0.01	0.10±0.03	0.23±0.04	0.34±0.02	0.15±0.02
	(79)	(137)	(60)	(45)	(97)	(95)	(31)	(41)	{174}	{334}	{174}	{334}
Mfn1&2	0.29±0.00	0.39±0.10	0.16±0.03	0.16±0.07	0.35±0.04	0.39±0.08	0.10±0.01	0.16±0.11	0.07±0.02	0.26±0.08	0.56±0.19	0.20±0.04
−/−	(19)	(28)	(10)	(9)	(25)	(28)	(7)	(9)	{55}	{129}	{55}	{129}
†Monte-Carlo simulations												
	0.27±0.07	0.47±0.03	0.14±0.05	0.12±0.04	0.40±0.04	0.40±0.04	0.09±0.04	0.11±0.04	0.06±0.02	0.14±0.00	0.32±0.04	0.21±0.03
	(1753)	(3493)	(1298)	(1176)	(1861)	(1888)	(512)	(629)	{2666}	{4268}	{2666}	{4268}

*Fu-Fi*: Fusion followed by fission; *Fi-Fu*: Fission followed by fusion; *Fu-Fu*: Fusion followed by fusion; *Fi-Fi*: Fission followed by fission; *Pd*: Paired; *UPd*: Unpaired.

The values in parentheses () are denoted as the absolute number of fission or fusion events for all the cells analyzed, while those in brackets {} are referred to the number of individual mitochondria observed.

ζ: Data were presented as the mean ± standard errors of values obtained from 14–65 mitochondria and 26–231 events totally.

† : Data were presented as the mean ± standard errors of values obtained from triplicate simulations at the same set of given parameters.

One of possible mechanisms for concurrent presences of four distinct patterns might be attributed to the stochastic nature of mitochondrial fusion and fission. To test this hypothesis, a Monte-Carlo simulation approach was employed and the mitochondrial dynamics was reproduced using “virtual” mitochondria experiencing random fusion and fission. At a typical set of initial parameters of *N* = 200, *T* = 200 s, *P^fu^* = 0.6, and *P^fi^* = 0.06, the frequency yielded respectively 0.27, 0.47, 0.14, and 0.12 (Table 1), which were consistent well with those from the measurements. Similar consistency was also found when the parameter set was systematically-varied (*data not shown*). Thus, the concurrent presences of distinct patterns of mitochondrial fusion and fission contribute to, at least partially, their intrinsic stochastic nature, which regulated mitochondrial dynamics. Again, the frequency of hetero-triggered pattern (0.74) was found to be higher than that of homo-triggered events (0.26) (*solid bar*, *P* = 0.069) (Fig. 4*A*), supporting the above observation.

### Paired fission or fusion event is dominated

Recent works suggested that only paired events for mitochondrial fusion and fission were found in the cells [4]. To further test this, we defined a paired event from a hetero-triggered pattern and an unpaired event from a homo-triggered pattern (Fig. 3). Thus, an ensemble of fission or fusion events observed were able to be segregated into four sub-groups of paired fission (*Pd-Fi*) and fusion (*Pd-Fu*) and unpaired fission (*UPd-Fi*) and fission (*UPd-Fu*) events. The results demonstrated that the paired events were dominant in mitochondrial dynamics, since much high fraction of paired events was observed in a Hela (0.79) (*open bars*) or MEF (0.79) (*hatched bars*) cell, as compared to that of unpaired events (0.21 or 0.21) (*P* = 0.001 or 0.000) (Fig. 4*B*). In contrast to the remarkable difference in occurrence frequency between *Fu-Fi* and *Fi-Fu* patterns, the fraction of paired or unpaired events yielded the same values between paired fission and fusion events in a Hela (0.39 and 0.40) or MEF (0.40 and 0.39) cell (Table 1), implying that the paired events were balanced each other no matter if it resulted from *Fu-Fi* or *Fi-Fu* pattern. Again, similar fractions were also obtained from the simulations with the values of 0.80 and 0.20 for paired and unpaired (*solid bars*, *P* = 0.028) (Fig 4*B*). Taken together, our results indicated that the paired events were dominated in the ensemble of fission and fusion events, but the unpaired events were not negligible. Note that the fraction of paired fission and fusion should not be necessarily equal to the summed fraction from hetero-triggered pattern since one event may be followed by more than one sequential event.

Mitochondrial dynamics were also tested using an accumulative frequency of paired or unpaired events. As exemplified in Figs. 4*C and 4D*, time course of accumulative frequency exhibited a straight line over entire duration and overlaid into a single line between fission and fusion events, suggesting that mitochondrial fission and fusion were balanced each other in a Hela (Fig. 4*C*) or MEF (Fig. 4*D*) cell. The slope of the line or the rate of occurrence, which determines how fast the occurrence is, was comparable between fission and fusion events (Table 2), as observed in yeast system [22]. Moreover, it was significantly higher for paired events ((1.52 and 1.59)×10^−3^ s^−1^ in a Hela cell and (1.34 and 1.42)×10^−3^ s^−1^ in a MEF-WT cell) than those for unpaired events ((0.24 and 0.41)×10^−3^ s^−1^ in a Hela cell and (0.25 and 0.42)×10^−3^ s^−1^ in a MEF-WT cell) (Table 2), imparting the confidence of domination of hetero-triggered pattern and activity of paired events.

**Table 2:** Summaries of slope of time-course of accumulative frequency.

	^ζ^Slope of time course (×10^−3^ s^−1^)					^ζ^Slope of time course (×10^−3^ s^−1^)			
Cell type	*Pd-Fi*	*Pd-Fu*	*UPd-Fi*	*UPd-Fu*	Cell type	*Pd-Fi*	*Pd-Fu*	*UPd-Fi*	*UPd-Fu*
Hela cell	1.52	1.59	0.24	0.41	MEF-WT cell	1.34	1.42	0.25	0.42
Hela cell+CCCP	1.68	1.43	0.14	0.14	MEF Mfn1 −/−	1.37	1.38	0.39	0.54
Hela cell+Nocodazole	1.70	1.65	0.20	0.21	Simulation	1.26	1.33	0.22	0.69

ζ: The slope α was obtained to fit the line using a simple linear equation, *AF* = α×*t*, where *AF* is the accumulative frequency at time *t*. The goodness-of-fit (*R* ^2^) yielded >0.810 except for one case of unpaired fission events in a Hela cell (0.24) where *R* ^2^ was 0.309 due to infrequent occurrence of events.

Not only the frequency and rate of occurrence of a fusion or fission event, but the lifetime and size of individual mitochondria could also be correlated to the distinct dynamics. Here a paired mitochondrion was denoted as the mitochondrion between two sequential paired events (that is, for *Fu-Fi* and *Fu-Fi* patterns) (*letter c* in Fig. 3*A* and *letters b and c* in Fig. 3*B* and *letter c* in Fig. 3*D*) and an unpaired mitochondrion was referred to the mitochondrion between two sequential same events (that is, for *Fu-Fu* and *Fi-Fi* patterns) (*letter b* in Figs. 3*C and 3D*). The lifetime or size of a mitochondrion measured individually was re-normalized by its maximum value for that cell and the data from different cells were pooled together for comparison. It was found that, in a Hela cell, the normalized lifetime was significantly lower while the normalized size was dramatically higher for paired events (0.04 and 0.28) than those for unpaired events (0.12 and 0.10), respectively (Table 1). Similar results were obtained in a MEF cell (0.07 and 0.35 for paired events and 0.13 and 0.14 for unpaired events, respectively) or from the simulations (0.06 and 0.32 for paired events and 0.14 and 0.21 for unpaired events, respectively) (Table 1), suggesting that the paired events be dynamically active with short lifetime and large size.

### Impact of membrane potential and cytoskeleton on mitochondrial dynamics

**Figure 5:**
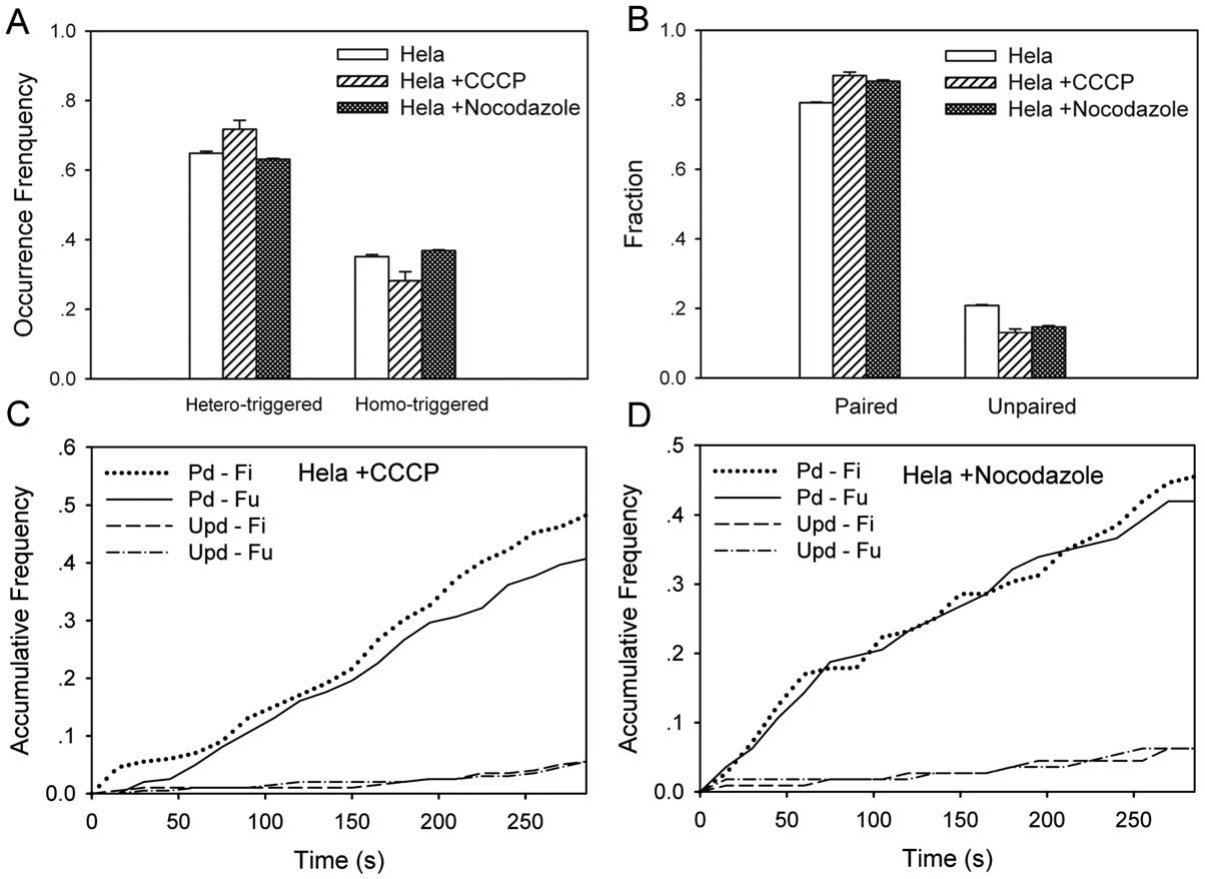
Multi-dynamic patterns in an intact or treated Hela cell. Comparisons of occurrence frequency of distinct patterns (*A*) and fraction of paired and unpaired events (*B*) in an intact (*open bars*), CCCP-treated (*hatched bars*), and nocodazole-treated (*solid bars*) Hela cell. Also plotted were the time courses of accumulative frequency of paired (*Pd*) or unpaired (*UPd*) fission (*Fi*) and fusion (*Fu*) events in a CCCP-treated (*C*) or nocodazole-treated (*D*) Hela cell up to *t* = 285 s. Data were presented as mean ± standard error (SE) measured from total 21–63 mitochondria and 121–216 events per cell.

Distinct patterns of mitochondrial dynamics observed are found to be associated with membrane potential. We thus tested if CCCP, a mitochondrial uncoupler by eliminating *H* ^+^ gradient across mitochondrial inner membrane [23]–[25] could affect mitochondrial dynamics. In the presence of 1 µM CCCP (Movie S4), the frequency of hetero-triggered pattern was slightly enhanced (from 0.65 to 0.72, *P* = 0.120) (*open and hatched bars* in Fig. 5*A*), especially for *Fu-Fi* pattern (from 0.24 to 0.30) (Table 1), and the fraction of paired events increased accordingly from 0.79 to 0.87 (*open and hatched bars* in Fig. 5*B*, *P* = 0.017), especially for *Pd-Fi* pattern (from 0.39 to 0.45) (Table 1), suggesting that the domination of paired events be attributed to the paired fission induced by CCCP. This was further observed from the estimations of normalized lifetime and size, where the size was reduced much (*i.e.*, from 0.28 to 0.19 for paired mitochondria) to form small-sized organelles whereas the lifetime remained the same (Table 1). The rate of occurrence for paired fission was enhanced (1.52 when CCCP was absent and 1.68 when CCCP was present) whereas that for paired fusion, unpaired fission, or unpaired fusion was reduced (1.59, 0.24, or 0.41 when CCCP was absent and 1.43, 0.14, or 0.14 when CCCP was present) (Fig. 5*C* and Table 2), indicating that the paired fission was activated when membrane potential was eliminated. Taken together, these results not only supported the above observation that four distinct patterns were present and the paired events were dominate and active for mitochondrial dynamics, but they also implied that the alteration of membrane potential *via* CCCP-treatment induced the variations in multi-patterned dynamics of mitochondrial fission and fusion.

Mitochondrial movement is associated with microtubule organization [16], [26], which might be related to mitochondrial dynamics of fission and fusion since it is necessary for two individual mitochondria to be brought sufficiently close to make a physical contact. Thus, we tested how the disruption of microtubule network affects mitochondrial dynamics when 10 µM nocodazole, a microtubule de-polymerizer, was added to Hela cells (Movie S5). It was indicated that the frequency of hetero-triggered pattern (0.63; *solid bar*) in a nocodazole-treated Hela cell was almost identical to that in an intact cell (0.65; *open bar*, *P* = 0.112) (Fig. 5*A*). Similar consistency was found in normalized lifetime and size (Table 1). The fraction and the rate of occurrence for paired events, however, were enhanced and those for unpaired events were reduced (*P* = 0.005) (Figs. 5*B and 5D* and Table 2), indicating that the de-polymerization of microtubule proteins activated the dynamics of paired events.

### Impact of mitofusin proteins

**Figure 6:**
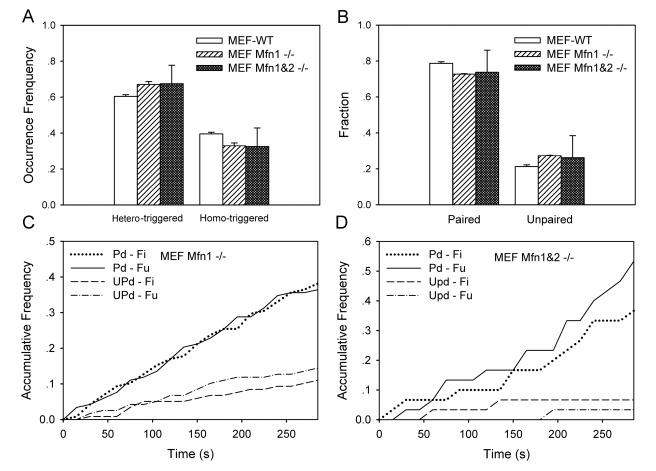
Multi-dynamic patterns in a wild-type or mutated MEF cell. Comparisons of occurrence frequency of distinct patterns (*A*) and fraction of paired and unpaired events (*B*) in a MEF-WT (*open bars*), MEF Mfn1 −/− (*hatched bars*), and MEF Mfn1&2 −/− (*solid bars*) cell. Also plotted were the time courses of accumulative frequency of paired (*Pd*) or unpaired (*UPd*) fission (*Fi*) and fusion (*Fu*) events in a MEF Mfn1 −/− (*C*) or MEF Mfn1&2 −/− (*D*) cell up to *t* = 285 s. Data were presented as mean ± standard error (SE) measured from total 16–65 mitochondria and 26–231 events per cell.

Mitofusin plays a critical role in regulating the balance of the fusion or fusion rate [27]. Next we tested the impact of mitofusin proteins on distinct patterns and paired events of mitochondrial dynamics. We first compared the mitochondrial fission and fusion in a MEF Mfn1 −/− cell (Movie S6) with those in a MEF-WT cell. It was found that the frequency of hetero-trigged pattern was enhanced (0.67, *P* = 0.025; *hatched bar* in Fig. 6*A*) but the fraction of paired events was reduced (0.73, *P* = 0.568; *hatched bar* in Fig. 6*B*) in a MEF Mfn1 −/− cell, suggesting that the occurrence of hetero-trigged pattern (especially for *Fu-Fi* pattern) is inversely correlated to the disappearance of paired events (Table 1). The normalized lifetime increased for unpaired events (from 0.13 to 0.23) while the normalized size remained the same (Table 1), implying that the activity of mitochondrial fusion was partially blocked. Moreover, the rate of occurrence was enhanced for the unpaired fission and fusion events as compared to those in a MEF-WT cell (Fig. 6*C*), implying that both the unpaired fission and fusion activities were accelerated when Mfn1 was knocked out.

We also compared the mitochondrial dynamics in a MEF cells that both copies of Mfn1 and 2 were knocked out (Movie S7) with those in a MEF-WT cell. Occurrence of mitochondrial fusion and fission in a MEF Mfn1&2 −/− cell was dramatically reduced to be respective 15–22 and 11–21 events per cell, as compared to those in a MEF Mfn1 −/− (respective 59–77 and 57–71 events per cell) and MEF-WT (respective 66–123 and 60–108 events per cell) cell, over observation duration of 210–435 s. This supported the fact that the fusion tended to diminish and the paired events intended to disappear in a MEF Mfn1&2 −/− cell [27]. By contrast, the fission rate also diminished in an equilibrium state where the mitochondria in the cell have been fragmented into small organelles with the smallest volume. Thus, not only the fusion rate declined but the fission rate also decreased to the minimum. Nevertheless, it is still worthwhile to analyze the dynamics of mitochondrial fusion and fission. Again, the frequency of hetero-trigged pattern was enhanced significantly (0.67, *P* = 0.568; *solid bar* in Fig. 6*A*) but the fraction of paired events was slightly reduced (0.74, *P* = 0.727; *open bar* in Fig. 6*B*) in a MEF Mfn1&2 −/− cell, supporting the inverse correlation between the occurrence of hetero-trigged pattern (especially for *Fu-Fi* pattern) and the disappearance of paired events (Table 1). While the normalized lifetime increased for unpaired events (0.26), as observed in a MEF Mfn1 −/− cell, the normalized size was exceptionally enhanced for paired events (0.56). This exception was probably due to the instability of mitochondrial dynamics in a MEF Mfn1&2 −/− cell, where the time course of accumulative frequency exhibited a fluctuating transition (Fig. 6*D*) and the linear fitting for unpaired events was even unable to be done due to the irregularity of the lines (Fig. 6*D*). Taken together, these results indicated that mitochondrial fission and fusion tended to be instable when Mfn1 and/or Mfn2 were abolished, even though the changes in the fractions of four distinct patterns upon the different treatments were not always obvious.

## Discussion

The goal of the current work was to develop a quantitative approach upon time-lapsed confocal microscopy and to understand the biological significance of distinct patterns of fusion and fission dynamics. Using the novel approach with the defined parameters, the dynamics of mitochondrial fission and fusion and the regulating mechanisms in a Hela or MEF cell were elaborately analyzed in the current work (Figs. 1, 2, 3, 4, 5, and 6 and Tables 1 and 2). We found that four distinct patterns of sequential events occurred concurrently in a living cell, *i.e.*, fusion followed by fission, fission followed by fusion, fusion followed by fusion, and fission followed by fission, and that the paired events yielded high frequencies with short lifetimes and large sizes of individual mitochondria. Our approach is distinct from the existing assays widely employed in mitochondrial biology and cell physiology. In those assays, the observations are based on the high-performance microscopy where living cells were treated with hypersensitive fluorescence-labeling biosensors, using photoactivatible green fluorescent protein and TMRE (tetramethylrhodamine ethyl ester) or red-fluorescent KFP (kindling fluorescent protein), to discriminate the individual mitochondria or mitochondria in two cells [4], [21]. While such the methods are highly sensitive in spatiotemporal resolution to monitor timely and local dynamics of mitochondrial fission and fusion, they are lacking a long-time and global data collection, which may result in the loss of global information of mitochondrial dynamics. Meanwhile, the quantitative analysis may not be specifically performed to describe the long time nature of total mitochondria in a living cell. By contrast, the novel approach reported here enabled us to visualize globally the life cycle of individual mitochondria in a living cell and to quantitatively analyze the dynamics of mitochondrial fission and fusion that is crucial to their biological functions. To assure that our approach can distinguish between true fusion or fission events and their simple apposition or departure of two closing mitochondria, we performed the supplementary measurements using the co-transfected Hela cells with mito-DsRED (*red*) and mito-PAGFP (*green*). These observations confirmed our results that we are able to identify the single mitochondria and visualize the mixing (and rupture indirectly) of inner mitochondrial compartments (Figs. S3 and S4).

Our findings provided new dynamic mechanisms of mitochondrial fusion and fission. In addition to the “kiss and run” pattern that a “transient” fusion triggered the following fission [21], which is similar to the *Fu*-*Fi* pattern in the current work, our results revealed three additional patterns of *Fi*-*Fu*, *Fu*-*Fu*, and *Fi*-*Fi*. In other words, we visualized the so-called “run and kiss”, “kiss and kiss”, and “run and run” patterns when mitochondrial fission and fusion events were monitored globally over entire cell (Fig. 2; *cf.* Movies S1 and S2), instead of a specific zone of the cell [21]. It was also found that the frequency of *Fu*-*Fi* pattern varied at 0.18–0.30 while that for the remaining three patterns summed up to 0.70–0.84 (Table 1), indicating that those additional patterns were statistically significant. This complicated dynamics was further confirmed from the viewpoint of paired events. While the occurrence of mitochondrial fusion and fission were considered as paired consecutive events upon a “kiss and run” pattern [4], the paired events from a “run and kiss” pattern and unpaired events from “kiss and kiss” and “run and run” patterns were also visualized in the current work (Figs. 2 and 3). Furthermore, paired mitochondria yielded shorter lifetime and larger size as compared to those for unpaired ones (Table 1), suggesting that the role of paired events are centered in mitochondrial function and cellular physiology. It should be noted that the four patterns are dynamic since the balance of mitochondrial size and number with time is retained over entire cell.

Our results also offered mechanistic insights on how mitochondrial dynamics is regulated and how it is associated with their biological functions. First, while the uncoupler CCCP, which uncoupled electron transfer along the respiration chain from oxidative phosphorylation and ATP synthesis [24], [25], is known to induce the continuous fission of mitochondria [23], our results were supportive in this regard, as seen in the enhanced frequency of *Fu*-*Fi* pattern and the increased fraction of paired fission events as well as the reduced size in the presence of CCCP (Table 1). Second, although mitochondria movement is known to be associated with microtubule organization that regulates the anterograde or retrograde movement of mitochondria along microtubule [18], [26], it was noticed that the de-polymerization of microtubule could not affect the fission and fusion dynamics of mitochondria [23]. Our data indicated that the de-polymerization of microtubule enhanced the rate of occurrence of paired events and reduced the activity of unpaired events. Interestingly, the summed fraction of paired and unpaired fusion events (0.49) was identical to that of paired and unpaired fission events (0.51) (Table 1), supporting the previous observation in the literature [23]. Finally, fusion proteins locating on outer membrane of a mitochondrion in mammalian cells are also associated with its biological function, as assumed for Mfn1 and Mfn2 to down-regulate mitochondrial networking when they were absent [27]–[29]. In a cell fusion system, mitochondria were 100% fused in a MEF WT cell and 94% in a MEF Mfn1 −/− cell but 0% in a MEF Mfn1&2 −/− cell [28]. This should turn out to be a prolonged lifetime when Mfn1 and/or Mfn2 were knocked out, similar to the observation of enhanced lifetime of unpaired events and increased frequency of fusion-triggered fission in the current work (Table 1). It was also found that both the fusion and fission activities were down-regulated when knocking out Mfn1 and Mfn2 proteins, resulted in an instable occurrence of mitochondrial fission and fusion (Fig. 6*D*). In addition to the agreement with the previous observations of mitochondrial function, our data presented new multiple patterns of action from the viewpoint of global dynamics of fusion and fission with the quantified lifetime and size.

Finally, we uncovered elaborately the multi-patterned dynamics of mitochondrial fission and fusion in a living cell, which provided an insight into mitochondrial evolution as well as the underlying regulations. Our results furthered the understandings in biological function of mitochondrial fission and fusion dynamics. This approach, combined with other assays such as fluorescence-labeling photoactivatible green fluorescent protein [4], [21], is applicable to elucidate the molecular mechanisms of mitochondrial fission and fusion.

## Materials and Methods

### Reagents

Mitotrack-CMX-ROS tracker was purchased from Cell Signaling Technology (Boston, USA), carbonyl cyanide 3-chlorophenylhydrazone (CCCP) and thymidine from Sigma-Aldrich (St. Louis, USA), and nocodazole from Alexis (Lausen, Switzerland). All other reagents were purchased from Beijing Chemical Reagents Company (Beijing, China) with the highest purity.

### Cell Culture

Human epithelial carcinoma (Hela) cells and wild-type mouse embryonic fibroblast (MEF-WT) cells were obtained from ATCC. Two MEF cell lines by knocking out Mfn1 alone (MEF Mfn1 −/−) and both Mfn1 and Mfn2 (MEF Mfn1&2 −/−) were described previously [27]. Four cell lines were respectively grown up in Dulbecco's modified Eagle medium (DMEM) (Hyclone, Logan, USA) supplemented with 10% heat-inactivated fetal bovine serum (Gibco, Tulsa, USA) and 1% of penicillin and streptomycin each (Hyclone, Logan, USA). Cells were cultured on a customer-made culture plate with a transparent window and treated with 1 µM Mitotrack-CMX-ROS for 20 min before laser confocal microscopic observation. Here the cells were maintained in the exact cell culture conditions (5% CO_2_ and 37°C) using a commercial temperature and CO2 control stage (Zeiss AIM-system 2104000112). For mitochondria uncoupling measurements, 1 µM CCCP were incubated with Hela cells immediately before observation. For microtubule depolymerization measurements, 10 µM nocodazole were incubated with Hela cells during observation [30].

### Visualization of mitochondrial fission and fusion

A two-photon laser scanning microscopy (Carl Zeiss LSM 510 Meta, Germany) was used to monitor the time courses of mitochondrial fission and fusion. Culture plate was placed on the stage of inverted microscope and time-lapsed images were recorded using a 60× oil immersed lens (Carl Zeiss Z500,Germany) at respective excitation and emission wavelengths of 524 and 600 nm. The spatial resolution varied in 0.05–0.14 µm/pixel when the scanning time interval of each frame was pre-set in a temporal resolution of 15–20 s per cycle. Time course of mitochondrial fission and fusion was monitored within 315–2320 s for a single cell. Total 2–6 cells were recorded for each case in two or three independent runs and the data of evolution dynamics of mitochondrial fusion and fission in two typical cells were presented (mostly, >150 events in one cell were identified spanning over 400 s). Acquired images were stored digitally and total 14–65 mitochondria and 26–231 events per cell were measured across entire duration. After image binarization, an individual mitochondrion was identified by setting the threshold to clearly isolate it from the neighbors (Fig. S2).

### Mitochondrial geometry and dynamics

A Matlab program was developed in-house to estimate the quantity, size, survival lifetime, accumulative frequency, and fission or fusion dynamics of individual mitochondria. Within an arbitrary *j*th cycle of cell slicing [*j* = 1, 2,,, *T*/*Δτ*, where *T* is the observation duration (∼315–2320 s) and *Δτ* is the time interval between two sequential cycles (∼15–20 s)], no fission or fusion was assumed to occur. Occurrence per cycle was counted for both fission and fusion events occurring in that cycle, from which the occurrence frequency, defined as the fraction in lumped number of occurrence between two sequential cycles, and the accumulative frequency, defined as the fraction of fission or fusion events up to the *j*th cycle in total number of events across entire duration, were then calculated. Quantity of individual mitochondria around splitting zone was estimated by identifying the contour of a single mitochondrion from sequential slices. Size and lifetime of individual mitochondria in 2D analysis, defined as the cross-section area and the duration spanning from its appearance to vanishment, respectively, were measured one-by-one in a Hela or MEF cell and then re-normalized by the respective maximum values for that cell, which enabled us to compare the data obtained from different cells.

### Three-dimensional reconstruction

Mitochondrial fission and fusion usually occur not only in the focus plane (*X*-*Y* plane) and but also along the direction perpendicular to the plane (*Z*-direction). Here a 3D reconstruction approach was developed to visualize the time-lapsed mitochondrial dynamics in *X*-*Y* plane and along *Z*-direction. Each slice was digitalized as a black and white image and the contour of a mitochondrion was identified. To filter out the background noise, a threshold of grey value was set for each cell (Figs. S1 *A*–*H*). Individual mitochondria in arbitrary three sequential slices were respectively colored in *red*, *green*, and *blue* from bottom to top, and then aligned together. This process was repeated from bottom (*Z* = 0) to top (*Z* = *H* where *H* is the maximum thickness of a cell). In 3D analysis, for simplicity, we presume that a mitochondrion has a uniform shape within one slice (the height is 1.0 µm) along *z*-axis. The size (here it is denoted as the volume of that mitochondrion) was calculated as cross-section area multiplied by slice height for each slice and the values for all the slices were summed up. Time course of 3D dynamics of mitochondrial fission and fusion was then obtained (Fig. S1 *A*′–*H*′. also seen in Movie S3).

### Simulation of mitochondrial dynamics

Monte-Carlo method was used to represent the dynamic process of mitochondrial fusion and fission. The basic point was to define a “virtual” microsphere organelle with a lower-limit volume to mimic a real mitochondrion. To further simplify the simulations, the 3D “virtual” organelle was projected into a plane to form a 2D organelle in the current work. Initially a given number of “virtual” organelles were placed into a rectangular plane with a defined size distribution and then allowed to undergo stochastic movements across the entire observation duration (*T*) to reproduce the evolution of mitochondrial dynamics. At a given moment *t*, the fate of an organelle was determined by comparing the pre-set probability of fusion (*P^fu^*) or fusion (*P^fi^*) with a random number (*ζ* ranging from 0 to 1) generated by computer, that is, the organelle fused with another one to form a big organelle when *P^fu^*>*ζ* or fragmented into two small ones when *P^fi^*>*ζ*. This happened simultaneously to all the organelles at time *t* and spanned across entire duration. Occurrence and accumulative frequencies of fusion and fission, and lifetime and size of individual organelles were calculated from the resulted evolution roadmap and re-normalized using respective maximum values for that test. Triplicate tests were performed and the average and standard deviation of dynamic parameters were estimated. Note that the fusion took place only when two neighboring organelle went into contact whereas the fission no longer happened when an organelle yielded a lower-limit volume. In the current work, the initial number of “virtual” organelles, *N*, ranged from 100 to 200, and initial size distribution of organelles followed an exponential distribution, the geometry of rectangular plane yielded 64×64 (or 45×45) µm, and the probabilities of *P^fu^* and *P^fi^* were pre-set to be 0.6 and 0.06 or 0.8 and 0.16, respectively, in different cases. The simulation duration, *T*, was given to be 200 s.

## Supporting Information

Figure S1 **Time-lapsed evolution of individual mitochondria in a Hela cell at** ***t*** **= 0 (** ***1^st^ row*** **), 12 (** ***2^nd^ row*** **), 24 (** ***3^rd^ row*** **), and 36 (** ***4^th^ row*** **) at two focused planes of** ***Z*** **_1_ = 7 µm (** ***1^st^*** **and** ***2^nd^ columns*** **) and** ***Z*** **_2_ = 8 µm (** ***3^rd^*** **and** ***4^th^ columns*** **) from the bottom (** ***Bar*** **= 2 µm).** Original *white and black* images were obtained from dual-photon laser scanning microscopy (*A*–*H*). *Colored* pictures were the 3D reconstructed ones where one set of three sequential images at *Z_j_*_−1_, *Z_j_*, and *Z_j_*_+1_ were colored by *red*, *green*, and *blue*, respectively, and superposed into a single image (*A′*–*H′*). This process was repeated using another set of three sequential images at *Z_j_*, *Z_j_*_+1_, and *Z_j_*_+1_,,, until all the images were reconstructed from bottom to top of the cell within entire duration. *Arrows* and *numbers* indicated typical events of mitochondria fission and fusion.
(TIF)

Figure S2 **Comparison of original (** ***left column*** **) and binarized (** ***right column*** **) segmentation of the typical images for MEF-WT cell (** ***1^st^ row*** **), CCCP-treated (** ***2^nd^ row*** **) Hela cell and MEF Mfn1 −/− cell (** ***3^rd^ row*** **), Bar = 4 µm.**
(TIF)

Figure S3 **Direct visualization of mitochondria dynamics in Hela cells co-transfected with mito-DsRED (** ***red*** **) and mito-PAGFP (** ***green*** **).** A photo-activation assay was used to visualize the mixting (*yellow*) and rupture of inner compartments of mitochodria. Briefly, cells were grown on a 14-mm diameter glass-bottom microwell dish. Laser light with a wavelengh of 405- or 413-nm was used for photo-activation of mito-PAGFP. Images were captured with a microscope (LSM 510, Carl Zeiss MicroImaging) using a 63×1.4 NA Apochromat objective. ROIs were selected and series of *z*-sections from top to bottom of the cell with a interval of 0.5–0.75 µm were irradiated using 405- or 413-nm light. The same intervals between optical sections were used for imaging. The increase in the amount of activated mito-PAGFP in non-activated mitochondria indicates that mitochondria fusion events were followed by the exchange of intra-mitochondria matrix contents (*arrows* show the sites where mitochondrial fusion happened). Mitochondrial fission events were also observed according to the division of a mitochondria into two or more parts (*arrowheads* denote the mitochondria with subsequent fission).
(TIF)

Figure S4 **Identification of individual mitochondria and fusion events by both binarization and photo-activation approaches.** Hela cells are co-transfected with mito-DsRED (*red*) and mito-PAGFP (*green*) and are activated under 405- (*red*, 1^st^ row) or 413-nm laser (*green*, 2^nd^ row). The mito-DsRED images are binarized (3^rd^ row), and merged with corresponding mito-PAGFP images (4^th^ row). *Blue* arrows indicate individual mitochondria, and *yellow* arrows indicate fusion events.
(TIF)

Movie S1 **Mitochondrial fission and fusion at the focal plane in a Hela cell.** *Red* or *green arrow head* indicated an impendent fusion or fission event. *Bar* = 5 µm.
(AVI)

Movie S2 **Mitochondrial fission and fusion at the focal plane in a MEF-WT cell.** *Red* or *green arrow head* indicated an impendent fusion or fission event. *Bar* = 5 µm.
(AVI)

Movie S3 **3D mitochondrial fission and fusion in a Hela cell.** *Red*, *green*, and *blue* indicated those mitochondria in arbitrary three sequential slices from bottom to top, respectively.
(AVI)

Movie S4 **Mitochondrial fission and fusion at the focal plane in a Hela cell treated by 1 µM CCCP.** *Red* or *green arrow head* indicated an impendent fusion or fission event. *Bar* = 5 µm.
(AVI)

Movie S5 **Mitochondrial fission and fusion at the focal plane in a Hela cell treated by 10 µM nocodazole.** *Red* or *green arrow head* indicated an impendent fusion or fission event. *Bar* = 5 µm.
(AVI)

Movie S6 **Mitochondrial fission and fusion at the focal plane in a MEF Mfn1 −/−cell.** *Red* or *green arrow head* indicated an impendent fusion or fission event. *Bar* = 5 µm.
(AVI)

Movie S7 **Mitochondrial fission and fusion at the focal plane in a MEF Mfn1&2 −/− cell.** *Red* or *green arrow head* indicated an impendent fusion or fission event. *Bar* = 5 µm.
(AVI)
