# Supplementary figures and images for: Multi-Patterned Dynamics of Mitochondrial Fission and Fusion in a Living Cell
Source: PLoS One. 2012 May 23;7(5):e19879. doi: 10.1371/journal.pone.0019879 (PMC3359327; doi:10.1371/journal.pone.0019879)

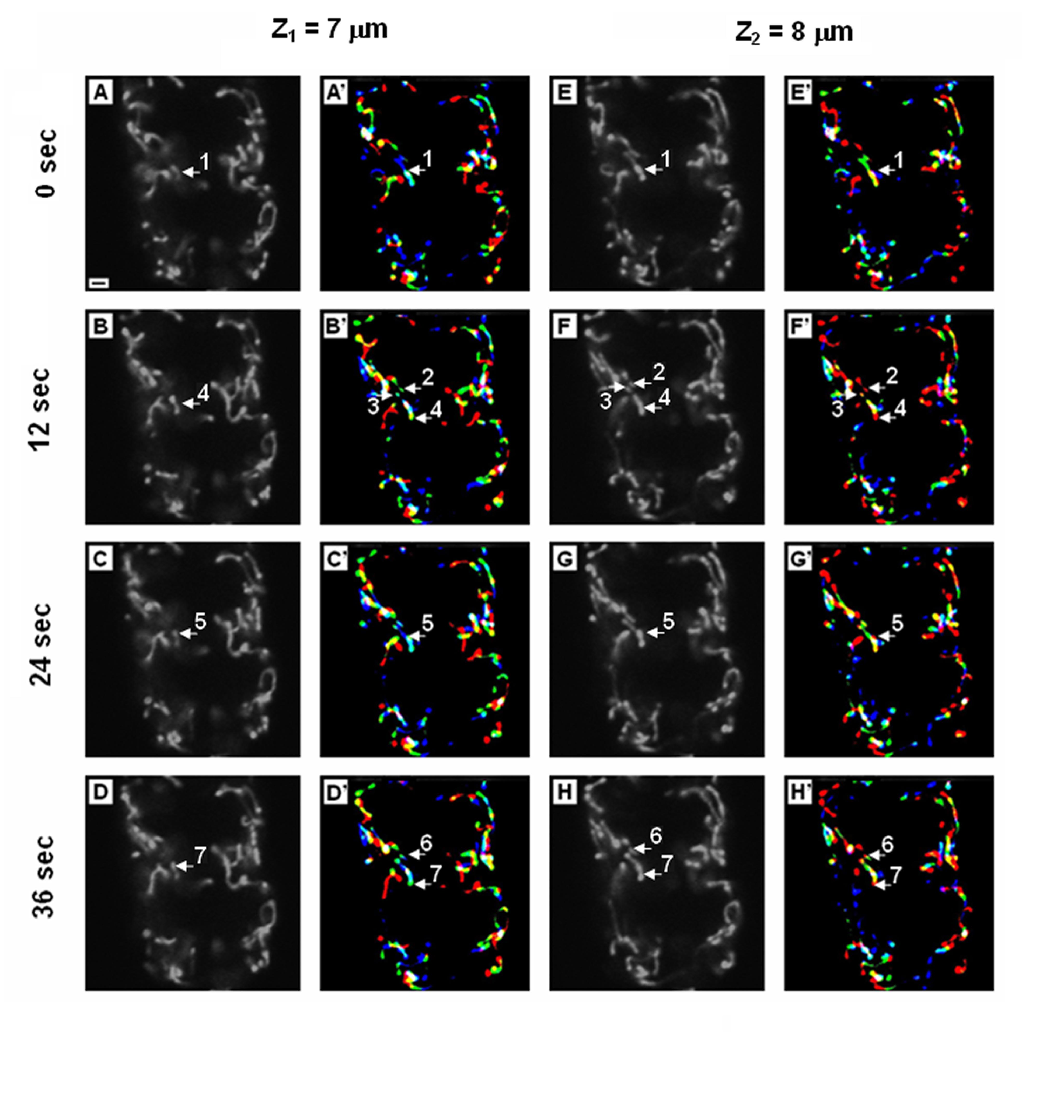

Supplement: Figure S1 — Time-lapsed evolution of individual mitochondria in a Hela cell at t = 0 ( 1st row ), 12 ( 2nd row ), 24 ( 3rd row ), and 36 ( 4th row ) at two focused planes of Z 1 = 7 µm ( 1st and 2nd columns ) and Z 2 = 8 µm ( 3rd and 4th columns ) from the bottom ( Bar = 2 µm). Original white and black images were obtained from dual-photon laser scanning microscopy (A–H). Colored pictures were the 3D reconstructed ones where one set of three sequential images at Zj −1, Zj, and Zj +1 were colored by red, green, and blue, respectively, and superposed into a single image (A′–H′). This process was repeated using another set of three sequential images at Zj, Zj +1, and Zj +1,,, until all the images were reconstructed from bottom to top of the cell within entire duration. Arrows and numbers indicated typical events of mitochondria fission and fusion. (TIF) [file pone.0019879.s001.tif]

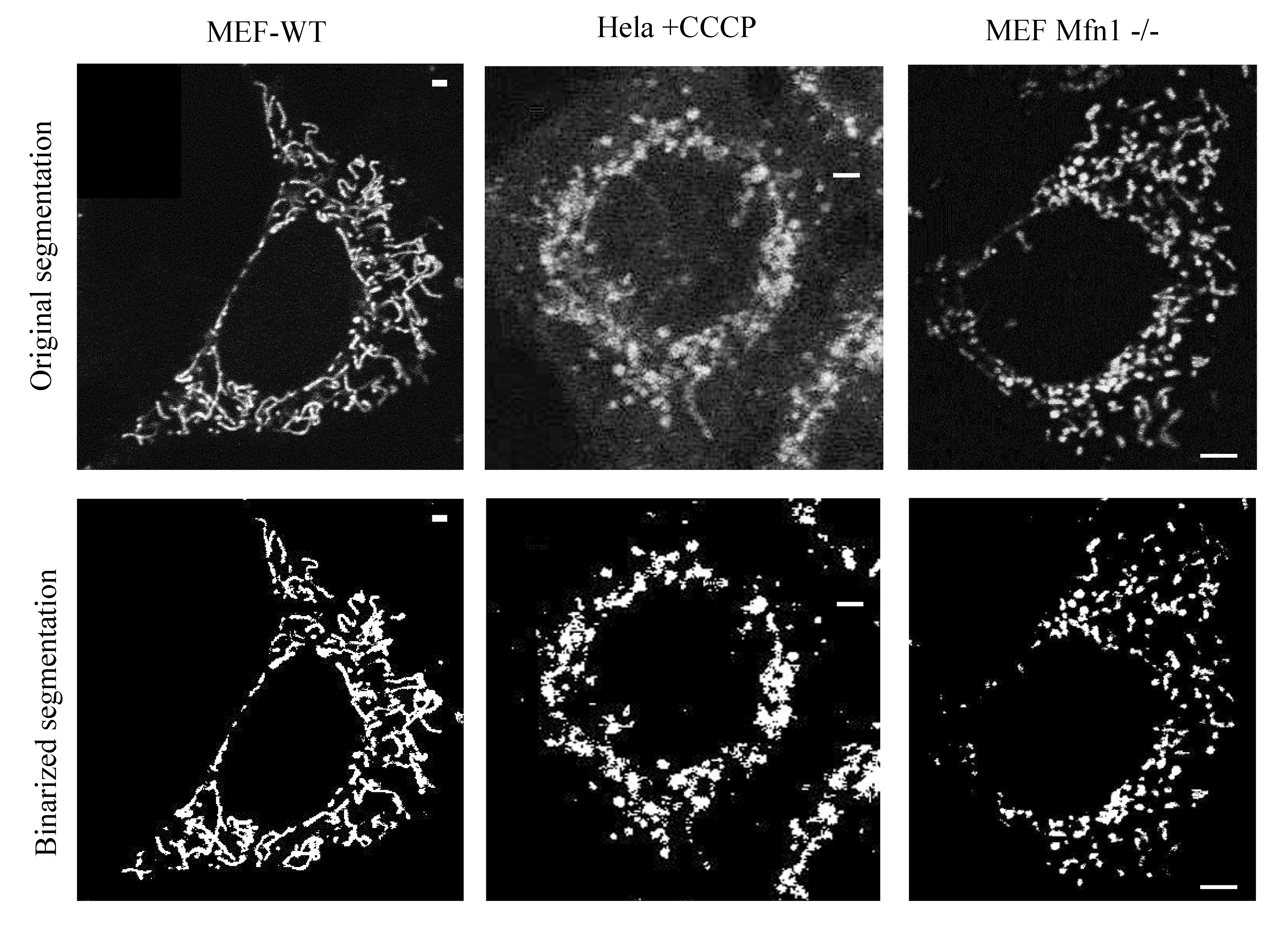

Supplement: Figure S2 — Comparison of original ( left column ) and binarized ( right column ) segmentation of the typical images for MEF-WT cell ( 1st row ), CCCP-treated ( 2nd row ) Hela cell and MEF Mfn1 −/− cell ( 3rd row ), Bar = 4 µm. (TIF) [file pone.0019879.s002.tif]

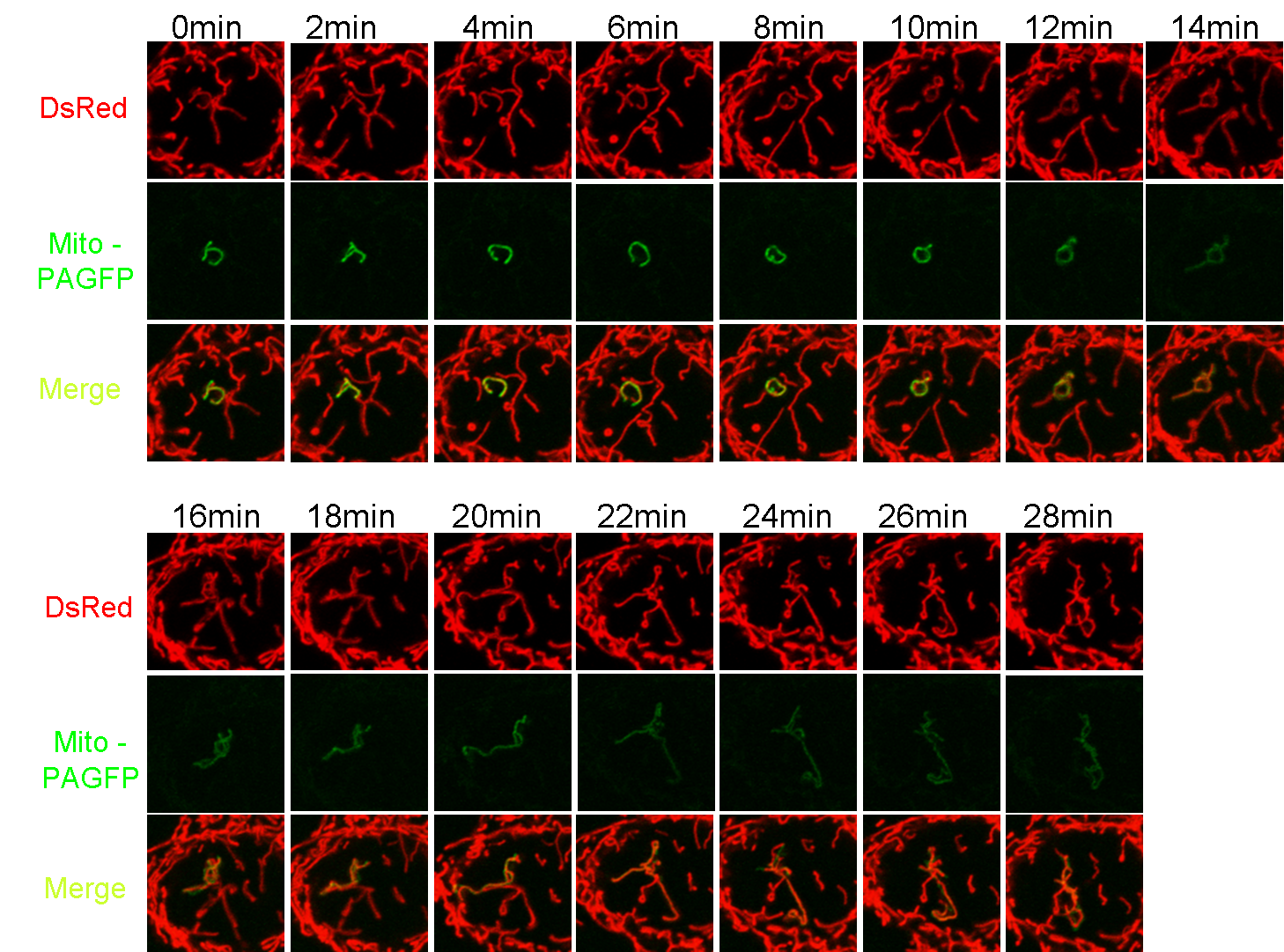

Supplement: Figure S3 — Direct visualization of mitochondria dynamics in Hela cells co-transfected with mito-DsRED ( red ) and mito-PAGFP ( green ). A photo-activation assay was used to visualize the mixting (yellow) and rupture of inner compartments of mitochodria. Briefly, cells were grown on a 14-mm diameter glass-bottom microwell dish. Laser light with a wavelengh of 405- or 413-nm was used for photo-activation of mito-PAGFP. Images were captured with a microscope (LSM 510, Carl Zeiss MicroImaging) using a 63×1.4 NA Apochromat objective. ROIs were selected and series of z-sections from top to bottom of the cell with a interval of 0.5–0.75 µm were irradiated using 405- or 413-nm light. The same intervals between optical sections were used for imaging. The increase in the amount of activated mito-PAGFP in non-activated mitochondria indicates that mitochondria fusion events were followed by the exchange of intra-mitochondria matrix contents (arrows show the sites where mitochondrial fusion happened). Mitochondrial fission events were also observed according to the division of a mitochondria into two or more parts (arrowheads denote the mitochondria with subsequent fission). (TIF) [file pone.0019879.s003.tif]

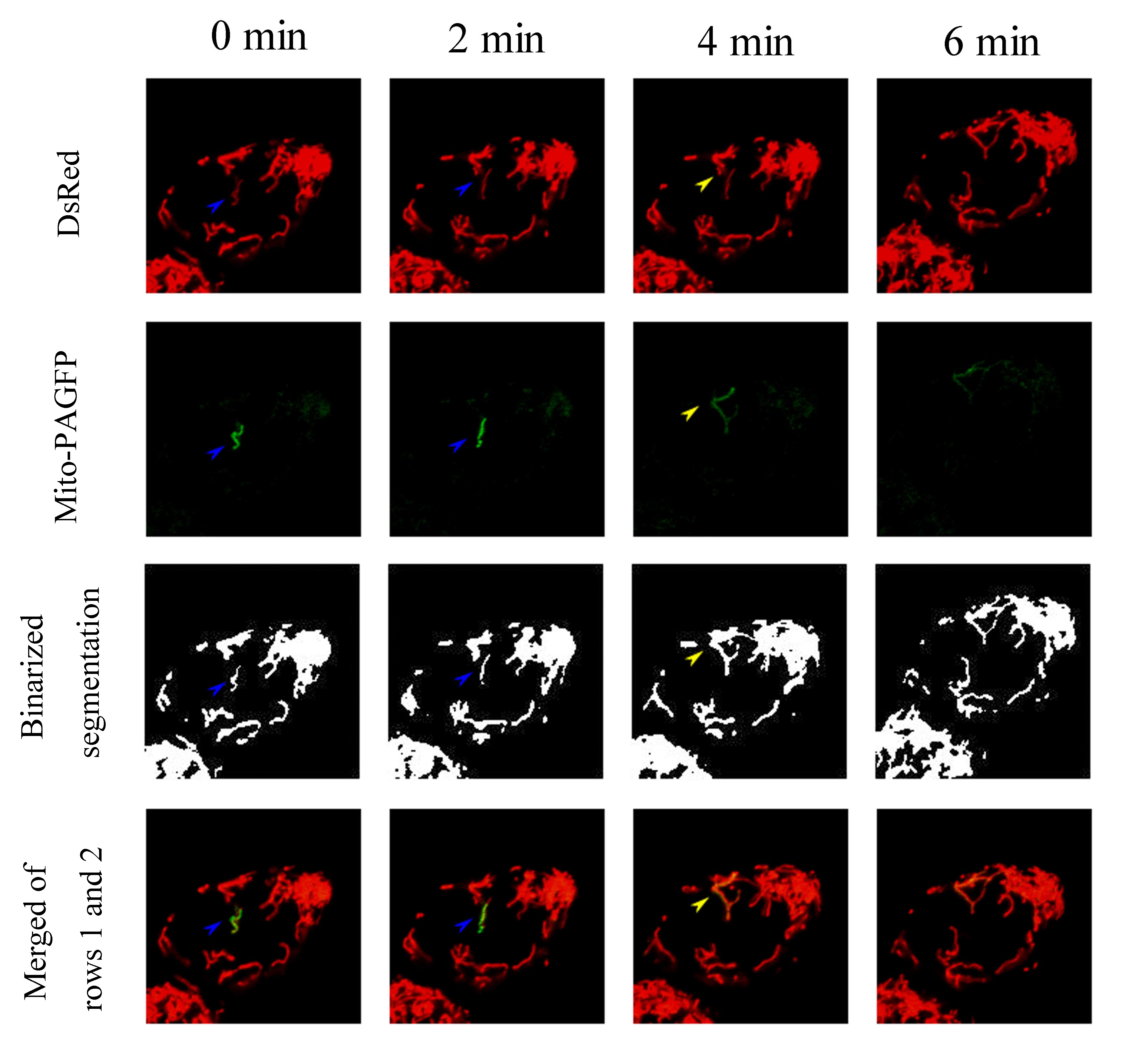

Supplement: Figure S4 — Identification of individual mitochondria and fusion events by both binarization and photo-activation approaches. Hela cells are co-transfected with mito-DsRED (red) and mito-PAGFP (green) and are activated under 405- (red, 1st row) or 413-nm laser (green, 2nd row). The mito-DsRED images are binarized (3rd row), and merged with corresponding mito-PAGFP images (4th row). Blue arrows indicate individual mitochondria, and yellow arrows indicate fusion events. (TIF) [file pone.0019879.s004.tif]
